# Physical activities at work and risk of musculoskeletal pain and its consequences: protocol for a study with objective field measures among blue-collar workers

**DOI:** 10.1186/1471-2474-14-213

**Published:** 2013-07-20

**Authors:** Marie Birk Jørgensen, Mette Korshøj, Julie Lagersted-Olsen, Morten Villumsen, Ole Steen Mortensen, Jørgen Skotte, Karen Søgaard, Pascal Madeleine, Birthe Lykke Thomsen, Andreas Holtermann

**Affiliations:** 1 National Research Centre for the Working Environment, Copenhagen, Lersø Parkallé 105, 2100, Copenhagen, DK, Denmark; 2 Department of Health Science and Technology, Aalborg University, Aalborg, Frederik Bajers Vej 7, 9220, Aalborg, DK, Denmark; 3 Department of Occupational Medicine, Køge Hospital, Lykkebækvej 1, 4600, Køge, DK, Denmark; 4 Institute of Sports Science and Clinical Biomechanics, University of Southern Denmark, Campusvej 5230, Odense M, DK, Denmark

**Keywords:** Diurnal measurements, Accelerometry, Musculoskeletal disorders, Physical exposure, Heart rate monitoring, Work ability, Productivity, Sickness absence, repeated pain measurement, DPhacto, Acti4

## Abstract

**Background:**

Among blue-collar workers, high physical work demands are generally considered to be the main cause of musculoskeletal pain and work disability. However, current available research on this topic has been criticised for using self-reported data, cross-sectional design, insufficient adjustment for potential confounders, and inadequate follow-up on the recurrent and fluctuating pattern of musculoskeletal pain. Recent technological advances have provided possibilities for objective diurnal field measurements of physical activities and frequent follow-up on musculoskeletal pain.

The main aim of this paper is to describe the background, design, methods, limitations and perspectives of the Danish Physical Activity cohort with Objective measurements (DPhacto) investigating the association between objectively measured physical activities capturing work and leisure time and frequent measurements of musculoskeletal pain among blue-collar workers.

**Methods/design:**

Approximately 2000 blue-collar workers are invited for the study and asked to respond to a baseline questionnaire, participate in physical tests (i.e. muscle strength, aerobic fitness, back muscle endurance and flexibility), to wear accelerometers and a heart rate monitor for four consecutive days, and finally respond to monthly text messages regarding musculoskeletal pain and quarterly questionnaires regarding the consequences of musculoskeletal pain on work activities, social activities and work ability for a one-year follow-up period.

**Discussion:**

This study will provide novel information on the association between physical activities at work and musculoskeletal pain. The study will provide valid and precise documentation about the relation between physical work activities and musculoskeletal pain and its consequences among blue-collar workers.

## Background

More than one million people in Europe have chronic musculoskeletal pain [1]. Musculoskeletal pain is the most prevalent cause of work ability loss, sick leave and early retirement from work in Europe [2-4]. Therefore, the burden of musculoskeletal pain on the individual and the society is tremendous [5,6].

It is well known that musculoskeletal pain is influenced by genetic factors [7], socio-economic factors [8], lifestyle [9] and individual perceptions [10]. However, high physical work demands are generally considered to be one of the main causes of musculoskeletal pain among workers [11]. Accordingly, workers with high physical work demands have the highest prevalence of musculoskeletal pain. Examples of these physical work demands are monotonous and repetitive arm movements, awkward body postures, prolonged standing, work with arms above shoulder height, and heavy lifting [11].

Numerous studies have investigated the association between physical work demands and musculoskeletal pain. However, several systematic reviews have concluded that the scientific documentation for a causal relation between high physical work demands and musculoskeletal pain is scarce [12-17]. The main critique of existing studies on the relation between physical work demands and musculoskeletal pain is the predominant use of self-reported measurements of physical work demands shown to have poor validity [18,19] and risk of confounding from age, gender and health measures like pain [20,21].

Moreover, most studies investigating the association between objectively measured physical work demands and musculoskeletal pain have used cross-sectional designs, making it impossible to draw conclusions about causal relations [20,22-25]. Furthermore, previous studies with objectively measured physical work demands are limited by short and often selected time windows [26-28] or measurements of physical demands in laboratory environments [18,29,30] lowering the external validity.

Another limitation of several current studies is insufficient adjustment for potentially confounding factors in the association between physical work demands and musculoskeletal pain. Particularly, possible confounders in this association are individual and socio-economic factors, lifestyle factors like leisure time physical activity and physical capacities [31,32]. Physical capacity such as muscle strength and endurance, flexibility and aerobic fitness has been related to risk of musculoskeletal pain [32-38]. Also low leisure time physical activity has been considered a risk factor for musculoskeletal pain [39], however studies present mixed results, possibly due to inadequate use of objective measurements [34,40]. Therefore, both of these potentially important confounders should be considered and measured objectively in studies of the association between work demands and musculoskeletal pain. Furthermore, previous episodes of musculoskeletal pain are often highly correlated with both the current physical work demands and the likelihood of future musculoskeletal pain, and a reverse causal relation with pain affecting the degree of exposure to physical work demands is likely. Finally, studies on the association between physical work demands and musculoskeletal pain are often limited by the lack of repeated follow-up on musculoskeletal pain. This is particularly a problem due to recall bias and fluctuating state of musculoskeletal pain [41]. Recent papers have demonstrated the advantage of repeating the measurements of musculoskeletal pain each month [42].

To summarise, a valid and reliable investigation of the risk for musculoskeletal pain from physical work activities should be based on objective diurnal field measurements of physical activities at work and leisure [19,43], using a prospective design with frequent registrations of musculoskeletal pain [41,42]. The study population should be large and homogeneous regarding socio-economic factors (e.g. including only blue-collar workers) to reduce confounding from socio-economic factors. Furthermore, appropriate measures of potentially confounding factors, like physical capacities, as well as previous episodes of musculoskeletal pain should be included.

Current technology and recent developments have provided equipment enabling measurements of physical activities at work and leisure over several consecutive days. Furthermore, frequent follow-up on musculoskeletal pain intensity is possible through a time saving, convenient and valid text messaging system [42].

### Purpose and hypotheses

The main aim of this paper is to describe the Danish PHysical ACTivity cohort with Objective measurements (DPhacto). The main aim of DPhacto is to investigate the association between objectively measured physical activities at work and frequent prospective measurements of musculoskeletal pain among blue-collar workers.

The main study hypotheses are:

– 1. High levels of physical activities at work increase the risk of developing musculoskeletal pain.

– 2. High levels of physical activities at work increase the risk for aggravation of musculoskeletal pain.

## Methods/design

This observational prospective study was approved by the Danish data protection agency and local Ethics Committee (H-2-2012-011).

### Study population

**Figure 1:**
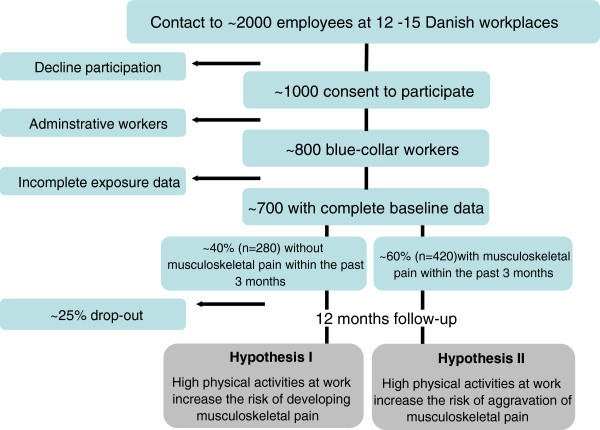
Flow diagram illustrating the recruitment of the study population, and its relation to the main hypotheses.

The study population is recruited from workplaces within the cleaning, transport and manufacturing sector in Denmark. Approximately 2000 employees from 12–15 companies in Denmark are contacted and invited to participate. The recruitment of the study population is illustrated in Figure 1.

**Figure 2:**
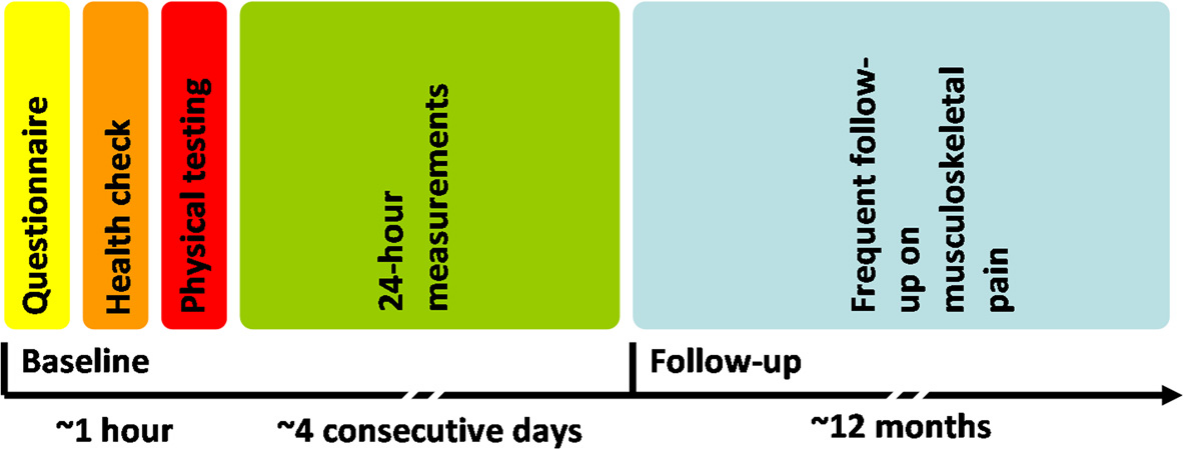
Timeline for the data collection and design of the study.

Eligible study participants are blue-collar workers with typically short education, low wage and a variety of physically demanding work tasks. Eligible workplaces give consent to allow measurements to take place during working hours. The participants are invited to fill in a short baseline questionnaire, participate in a health check and a physical test session. Furthermore, participants are invited to wear a 24-hour measurement kit for four consecutive days. Finally, participants are asked to reply to text messages each month for a year concerning musculoskeletal pain and musculoskeletal-related sickness absence, and a questionnaire each quarter for one year concerning consequences of musculoskeletal pain (e.g. productivity and work ability). The overall design and timeline of the study are shown in Figure 2.

Participants not consenting to participate in the health check and physical test session or 24-hour measurements are invited to consent to fill in the baseline questionnaire only. After participation, all volunteers receive a report showing and interpreting their results in relation to the overall population of participants.

### Data collection

Data will be collected from spring 2012 till spring 2014. The baseline data collection of questionnaires, health check, physical test and the four consecutive 24-hour measurements will start at the first workplaces in spring 2012 and at the last workplaces in the beginning of 2013.

### Baseline objective measurements and questionnaires

Objective measurements of height, body mass, hip and waist circumference, percentage body fat and blood pressure are collected during the health check.

#### Objective measurements of physical capacity

Physical capacity measurements are collected during the physical testing session. All measurements are performed by trained clinical personnel (physiotherapists, physiologists and exercise physiology students). The specific physical capacity measurements are chosen based on three main criteria: 1) reasonably high validity with more rigorous capacity measurements [44-46] 2) relation with physical work capacity [36,47] and 3) suitability as field measures – low performance requirements for the participants (compared to i.e. maximal capacity tests), low costs and easy to move around. The measurements will be used in the analyses if they play a role in the relation between physical work demands and muscle pain. The measurements are:

#### Maximal oxygen uptake

A submaximal cycle ergometer test is conducted on an Ergomedic 874 E cycle ergometer (Monark AB, Varberg, Sweden) [48]. The initial power output is estimated based on age and estimated fitness, and is typically 60 to 90 W at the predetermined cadence of 60 revolutions/min. Heart rate is measured with a handheld pulse oximeter (Nellcor OxiMax N-65 , US) attached to the fingertip during the test. If the heart rate is less than 110 beats/min after the first minute, power output is increased with the goal of achieving a heart rate at or above 60% of the estimated maximal heart rate capacity and at least 120 beats/min. If heart rate has reached a steady state, defined as less than 5 beats/min change from the 5^th^ to the 6^th^ min, the test is terminated and heart rate registered. Otherwise, the participant continues cycling until a steady heart rate is reached. The maximum duration of cycling is 10 min. Subsequently, the power output and corresponding heart rate are used to estimate maximal oxygen uptake using the Åstrand-Rhyming nomogram with correction for age and gender [48].

#### Maximal hand grip strength

Test of maximal voluntary isometric hand grip strength is performed for hand grip flexion of the dominant hand according to a standardised procedure [45]. Participants are instructed to gradually build up force over 5 s, to keep the maximal force for another 2 s, and to finally reduce force slowly. The test is performed at least three times. If the third test results in more than 5% higher force than either of the previous two tests, a fourth test is performed. A maximum of five tests will be performed. Strong verbal encouragement is given during the test.

#### Back extension endurance

A standardised isometric back extension endurance test (Biering-Sorensen test) is performed [38]. Participants are lying prone on a sloping board with the head highest (70 x 40 x 15 cm). The participants hold their upper body in a horizontal position with the arms folded across the chest and with a hip flexion of approximately 12°. The position is held for as long as possible, but to a maximum of 360 s [49].

#### Flexibility test

The finger-to-floor method is used [50]. The participants stand on a 30 cm high box and bend forward while pressing a horizontal measurement slide downwards. Sagittal flexibility is defined as the distance from the fingertips to the box level in the fully flexed position.

#### Self-reported measures

A structured self-administered questionnaire with validated measures is applied at baseline. The questionnaire involves socio-demographic measures (e.g. age, gender, height, weight, ethnicity, country of birth, employment status); lifestyle and health (e.g. smoking, alcohol consumption, medicine use, sleeping behaviour, general health [51]); Standardized Nordic Questionnaires for the analysis of musculoskeletal disorders [52]; musculoskeletal pain-related sickness absence [53]; productivity loss and interference 7with daily work activities [54]; the single-item self-evaluated overall work ability from the Work Ability Index [55,56], and perceived physical exertion during work [57].

### Objective measurements of physical activity during work and leisure

#### Procedure

Participants are asked to wear four accelerometers and one heart rate monitor for a minimum of four consecutive days. Participants are instructed to carry the equipment during all activities during the measurement period and to perform one reference measurement in upright stance of 15 s every day. The participants are also instructed to remove the equipment if it causes itching or discomfort resulting in e.g. disturbed sleep. Participants are equipped with extra plaster if the equipment unintentionally falls off and instructed to replace the items. The four days of measurement include at least two working days, and optimally two days off work. Many blue-collar workers in Denmark do not follow a typical Monday-Friday work schedule, but have days off during the week and may instead work during weekends. Therefore, specific collection days will vary between workplaces and workers. Activities during leisure time will be included as mediators/confounders in the main hypotheses. During the measurement period, participants are asked to fill in a short diary concerning working hours, leisure time, sleep and time of reference measurement. At the end of the four-day data collection, the equipment is collected.

#### Instrumentation and adhesion

The accelerometers are of the model ActiGraph GT3X + (Actigraph, Florida, U.S.A). The Actigraph is a triaxial accelerometer enabling estimation of energy expenditure and number of steps (ActiLife version 5.5). Actigraph (19 g, 4.6 × 3.3 × 1.5 cm) is waterproof, and can be worn during showering. Raw acceleration data is collected and stored for up to 10 days of measurement. The accelerometers are initialized for recording and data downloaded using the commercial manufacturer’s software (ActiLife version 5.5). Additional advanced analysis of Actigraph recordings enables estimations of activity types and body positions through our own developed software Acti4: Activity: type-duration-variation-posture (Acti4).

The accelerometers are fixed by tape (3 M, Hair-Set, double sided adhesive tape and Fixomull, BSN medical and flexifix) at four anatomical sites. For detection of upper arm elevation (flexion and abduction), an Actigraph is placed 3 cm distal to the deltoid insertion of the dominant hand [58,59]. For detection of the inclination of the upper back, an Actigraph is placed with the upper border of the accelerometer at the level of the T1/T2 of the upper back. Placement of the Actigraph on the hip is near the upper point of the iliac crest at the right side. The placement of Actigraph on the right thigh is medial between the iliac crest and the upper border of the patella.

Electrocardiography is monitored with Actiheart (Camntech, Cambridge, United Kingdom). The Actiheart measures uniaxial accelerometry and electrocardiography (ECG) enabling estimation of heart rate, heart-rate-variability (HRV) and energy expenditure for up to 72 hours [60,61]. The small size (10 g, 18.8 cm by length) and use of standard biocompatible ECG electrodes ensure that the Actiheart is securely but comfortably attached to the chest. Actiheart is water resistant and compatible with daily activities. ECG is measured electronically with a sensitivity of 0.250 mV, and HRV is calculated by the stored inter beat intervals (time interval between R’s in the QRS complexes). The Actiheart is placed at the apex of the sternum with a horizontal wire to the square clip at the left intercostals space, at the level of the 6th and 7th costae found to be the position least affected by artefacts. The Actiheart is previously used on workers with physical demanding work, and validated for measurements of HRV [60].

### Prospective follow-up on development of musculoskeletal pain (primary outcome) and its consequences (secondary outcomes)

#### Questionnaires

A text messages based method is applied as a monthly follow-up on the intensity of musculoskeletal pain and musculoskeletal pain-related sickness absence [42]. Each month, the participants receive three questions on their mobile phone during the entire 12 months follow-up. The participants receive the questions on Sundays, with a reminder on Mondays. The questions covering the primary outcomes of the study are: “On a scale of 0–10, grade the worst pain you have experienced in your neck/shoulder within the past month? (0 = no pain, 10 = worst possible pain)”, “On a scale of 0–10, grade the worst pain you have experienced in your lower back within the past month? (0 = no pain, 10 = worst possible pain)” and the question covering one of the secondary outcomes is “Within the past month, how many days have you been absent from work due to pain in muscles or joints? Reply from 0–31 days”. In addition three text messages are applied as a quarterly follow-up regarding current work ability, the influence of musculoskeletal pain on physically heavy work activities and social activities (all rated on a scale from 0–10) covering secondary outcomes.

#### Exclusion criteria

Participants are excluded from testing of physical capacity measurements (i.e. aerobic capacity or back extension endurance test) if they are diagnosed as hypertensive, have a blood pressure measurement with diastole ≥160 mmHG or systole ≥100 mmHg, angina pectoris, previous herniated disc or daily use of heart or lung medicine. Fever on the day of testing will exclude participants from the physical capacity and diurnal measurements. Pregnancy will exclude from participation in the study. Furthermore, participants are asked if they have considerable musculoskeletal pain in the back on the test day. If they do, they are excluded from the back extension endurance test. Participants are excluded from the diurnal measurements if they report allergy to bandages or adhesives.

### Data analyses

The objective measurements of the physical activities at work based on the Actigraph and Actiheart recordings will be analysed by first the original commercial software of the equipment and specially developed custom build software for estimating physical activity types, duration, variation and body postures Acti4 [59]. Variables are:

– Body and arm position (e.g. standing, sitting, lying, upper body inclination, arms above shoulder height).

– Activity types (e.g. walking, running, stair climbing, bicycling).

– Heart rate and intensity during different activities.

– Heart rate variability.

– Number and frequency of steps.

– Physical activity energy expenditure.

Validation of the measurement of sitting, standing, walking, running, stair climbing and bicycling have been conducted in during semi-standardized and free-living conditions and shown specificity and sensitivity as high as 99% and a 100% [59]. Furthermore, software for the measurement of temporal and structural variability [61,62] is being developed by our laboratories and will potentially supply more variables to the list at the time of analyses.

### Statistical analyses

Hypotheses 1 and 2 will be tested for each pain site separately. All analyses will be performed using SAS® version 9.2 or later for the Windows platform.

Analyses of Hypothesis 1: High levels of objectively measured physical activities at work increase the risk of developing musculoskeletal pain:

The test of Hypothesis 1 will be based on analyses of the effect of the physical activity on the incidence rate of first occurrence of pain (pain intensity > 0) at the specific site among participants who are free of pain (i.e. pain intensity = 0) at the specific site at baseline. The effect on the incidence rate will be modelled using the Cox regression model applied on grouped event-time data [63]. The assumption of proportional hazards will be evaluated graphically, and the potential consequences of observed deviation from proportionality will be commented on.

Analyses of Hypothesis 2: High levels of objectively measured physical activities at work increase the risk for aggravation of musculoskeletal pain:

The test of Hypothesis 2 will be based on analyses of the effect of the physical activity on the repeated measurements of the level of pain at the specific site among participants with pain (i.e. pain intensity > 0) at the specific site at baseline.

The analyses will be based on a mixed-model-repeated-measurement (MMRM) analyses of change from baseline with the effect of the exposure variable interacting with the time point (month), using the observed cases and an unstructured variance-covariance matrix. The MMRM analyses will be performed without as well as with the baseline pain level included as a covariate interacting with the time point.

For both hypotheses, the effects of the different measurements of physical activity will be investigated using linear splines in the regression models, thereby assuming that the association is linear within the intervals between some pre-specified knots, while allowing the slope to change at the knots. Thus, a continuous association between the outcome and the exposure is assumed while allowing the association to be non-monotonic [64]. The knots are placed so that sufficient amount of statistical information is available in each interval. It will be tested whether a simple linear association may be assumed and it will be investigated whether a more flexible, linear spline (that is, adding additional knots) will provide significant additional information indicating strong deviations form a simple dose–response relationship. Potential confounders (such as physical capacity and leisure time physical activities) will be considered on an exploratory basis.

### Sample size calculations

The sample size calculations are based on the primary outcome-exposure relation which is the unadjusted association between LBP and the working time spent bent forwards >60 degrees among subjects with baseline exposures above a threshold of 5 minutes per working day, assuming a linear association with the exposure in the Cox regression as well as in the MMRM analysis. The sample size calculations corresponding to power of 80% for detection of either of the two hypotheses are based on the use of two independent, two-sided tests, each at the 2.5% significance level in unadjusted analyses.

We have not been able to identify any data or studies with regression analyses of changes in pain corresponding to a certain amount of work time spent with one activity to guide our estimation of the effect size of exposure on pain. However, we have reviewed prospective studies using categorized exposure variables to estimate a realistic order of magnitude of the potential effect. For the MMRM analysis of subjects with pain at baseline, the effect of the exposure on change in pain is given per one standard deviation’s difference in the exposure variable in our pilot population (0.25 hours) and the corresponding expected change in pain is the lowest possible within a reasonable study population size. That is, we aim to be able to detect a change in pain of 0.65 on a scale from 0–10 per 0.25 hour’s difference in average daily working time spent bent forwards >60 degrees. For the Cox regression we assumed a HR of 1.4 per 0.25 hours of exposure. Among the individuals above the 5 minutes threshold in our pilot study, this assumption corresponds to an average HR of 1.6 for individuals above the median exposure compared to individuals below the median exposure. This HR corresponds to levels previously found in studies of the effect of high vs. low exposures on pain [65]. The required sample size for the analysis of the change in pain was calculated as the sample size needed in a simple linear regression model of the change in pain at 12 months on the baseline exposure. The application of a MMRM on all of the repeated pain measurements will give a higher power compared to the simple linear regression model applied to the final measurement, thus the power of the study is expected to be higher than requested.

Based on data on three repeated monthly LBP measurements collected from a subsample of the current study sample, the variance of the change in pain measurements at later time points was estimated to be twice the variance of 7.35 seen in the observed LBP at 2 months post baseline for subjects with LBP at baseline. The variability in the exposure variable, working time with back bent forwards >60 degrees (measured in hours), was based on data from a pilot study of a working population comparable to the one aimed to be recruited for the current study. The calculations of the variability in the exposure was made separately within each of the two subpopulations applicable for hypothesis 1 and 2; that is, workers with LBP = 0 at baseline and workers with LBP > 0 at baseline, respectively. Among the participants with a measurement of baseline pain in the pilot study, 57% had LBP > 0 at baseline. Among the participants with LBP = 0 at baseline, the exposure variable was missing for 13%; 72% of the rest had an exposure above the threshold of 5 minutes per day and among these, the variability of the exposure as measured by the corrected sum of squares of deviations divided by number of subjects (CSS/N) was 0.128 (corresponding to a standard deviation of 0.36 for the exposure variable measured in hours). Among the participants with LBP at baseline, the exposure variable was missing for 8%; 75% of the rest had an exposure above the threshold and among these, the variability of the exposure was CSS/N = 0.0693 (corresponding to a standard deviation of 0.26 for the exposure variable measured in hours).

Based on the above estimated variability in the exposure, a total number of at least 52 new events of LBP are required among the subjects with exposure above the threshold of 5 minutes per day and LBP = 0 at baseline, and measurements of pain at 12 months follow up are required for at least 205 subjects with exposure above the threshold and LBP > 0 at baseline.

Previous papers indicate that a 1-year incidence for any LBP of 20-40% can be expected for individuals without LBP at baseline [66,67]. The reports of an incidence of 20% are based on a 1-year recall, and may therefore underestimate the true incidence as indicated from a recall study [42]. Therefore, a 1-year LBP incidence of 30% is assumed in the current study. Assuming a drop out of 25% and a 72% prevalence of exposure above the threshold of 5 minutes per day, we thus need at least 269 subjects with LBP = 0 at baseline. For the analysis of the change in pain, we need 367 subjects with LBP > 0 at baseline assuming a drop out of 25% and a 75% prevalence of exposure above the threshold. Based on the LBP prevalence of 57% in the pilot study, a total of at least 643 subjects with baseline measurements of both exposure and LBP are needed for this study, corresponding to 276 subjects with LBP = 0 and 367 subjects with LBP > 0. Therefore, we aim at recruiting around 700 subjects with baseline measurements of both exposure and LBP.

### Missing data handling

At least one workday with objective measurements per participant will be required for data analyses. We will investigate predictors for missing data (i.e. non-participation at all, missing data on objective measurements or missing data on follow-up pain measurements). In the Cox regression, participants will automatically be censured at the first missing data measurement during follow-up – no matter the reason for the missing data (i.e. spot-like missing or complete drop-out).

For the MMRM model, the observed-cases approach will be used, that is, all non-missing pain measurements will be included and no imputations will be performed, since this approached is unbiased in MMRM model under the assumption that there are no informative missing, that is, when all the available data on the subject is taken into account then the fact that an observation is missing does not in it-self indicate anything about the true (unobserved) pain level at the given time point.

## Discussion

Overall, the DPhacto study will extend the knowledge on the association between detailed measurements of physical activities at baseline and the subsequent risk of musculoskeletal pain and its consequences. The study provides data to conduct differential evaluation on the consequences of physical work demands among individuals with and without musculoskeletal pain at baseline, respectively. Finally, the study results will potentially improve the scientific evidence for prevention strategies towards musculoskeletal pain. Current strategies to prevent musculoskeletal disorders have in many cases failed to actually *prevent* musculoskeletal disorders [68]. For example, work reorganization has been conducted, however with improper reorganization of work tasks [69]. This study will disentangle different body postures and movements and their contribution to the risk of musculoskeletal pain and thereby more precisely specify risky postures, activities or durations of activities to more precisely guide preventive activities.

### Strengths and limitations

This present study introduces several important strengths to add to the current knowledge on the association between physical activities at work and during leisure time and risk for musculoskeletal pain and its consequences. Objective recordings of physical activity will be made at the workplace without interference from experimenters. One can thus consider that the recorded measurements will represent genuine information about the specific physical workload. Objective measurements of physical activities will be made at work and during leisure enabling a separation of leisure time physical activity from work. Finally objective measurements of physical activity types at work constitute a valid and reliable measure that has not previously been used in prospective studies. The objective measurements are conducted over several consecutive days, which improve the representativeness of the measurements and allows for thorough confounder control for leisure time activities. Furthermore, the objective measurement allows for the analyses for a dose–response relationship between physical work demands and musculoskeletal pain. The prospective follow-up on repeated measurements of musculoskeletal pain reduces recall bias and allows for event-time analyses. The sample size of the study permits separate analyses of pain-free individuals and individuals with musculoskeletal pain at baseline, respectively. Finally, objective measurements of the physical capacity allow for investigation of their mediating role on the association between physical work demands and musculoskeletal pain.

The study also faces some challenges. First, objective measurements of the loads during lifting, pushing and pulling is not possible with the small, discrete measurement devices used in this study. Therefore, these loads are self-reported. Self-reported values of these measures are likely to be related to the subject’s level of pain, i.e. individuals with higher pain levels may perceive their work tasks to be more heavy. Therefore, the association between concurrent exposures and pain may be biased, however, the consequence for the association between the exposure and prospective follow-up in pain remains unclear. Second, recruitment through workplaces and requirements of conducting all measurements during working hours may introduce selection bias among the companies, i.e. primarily companies with higher resources may choose to participate, but only workplaces with primarily blue collar workers are recruited and thus the workplaces are primarily relatively low profit businesses with tightly scheduled productions. In the same line, an expected participation rate of 60% among the eligible employees may introduce selection bias among the participants. An attempt to collect self-reported descriptive data on employees not participating in the objective physical activity measurements will be made. Finally, individuals’ mental health as well as somatising are not measured but may play a role in the relation between physical activities at work and musculoskeletal pain.

## Competing interests

The authors declare that they have no competing interests.

## Authors’ contributions

AH conceived the research idea. AH and MBJ discussed and wrote the initial protocol as well as the design of the study. MBJ was responsible for drafting the paper. AH was responsible for application for the ethical committee. MBJ, AH and BLT discussed the analyses and BLT conducted the power calculations and wrote the analyses section. AH, MKL and JS developed the method for the identification of physical activities from the actigraph measurements and JS the software Acti4. JL-O, MV, OSM, KS, and PM participated in and supervised discussions about methods for measurements and analysis. All authors have read and commented on the draft version and approved the final version of the manuscript.

## Pre-publication history

The pre-publication history for this paper can be accessed here:

http://www.biomedcentral.com/1471-2474/14/213/prepub
